# Comparison of the Intracellular Trafficking Itinerary of CTLA-4 Orthologues

**DOI:** 10.1371/journal.pone.0060903

**Published:** 2013-04-02

**Authors:** Satdip Kaur, Omar S. Qureshi, David M. Sansom

**Affiliations:** The MRC Centre for Immune Regulation, School of Immunity and Infection, University of Birmingham Medical School, Birmingham, United Kingdom; INRA, France

## Abstract

CTLA-4 is an essential inhibitor of T cell immune responses. At steady state, most CTLA-4 resides in intracellular compartments due to constitutive internalisation mediated via a tyrosine based endocytic motif (YVKM) within the cytoplasmic domain. This domain is highly conserved in mammals suggesting strong selective pressure. In contrast, the C-terminal domain varies considerably in non-mammals such as fish, *xenopus* and birds. We compared the ability of the C-terminus of these species to direct the trafficking of CTLA-4 with human CTLA-4. Using a chimeric approach, endocytosis was found to be conserved between human, *xenopus* and chicken CTLA-4 but was reduced substantially in trout CTLA-4, which lacks the conserved YXXM motif. Nevertheless, we identified an alternative YXXF motif in trout CTLA-4 that permitted limited endocytosis. Post-internalisation, CTLA-4 was either recycled or targeted for degradation. Human and chicken CTLA-4, which contain a YVKM motif, showed efficient recycling compared to *xenopus* CTLA-4 which contains a less efficient YEKM motif. Specific mutation of this motif in human CTLA-4 reduced receptor recycling. These findings suggest evolutionary development in the endocytic and recycling potential of CTLA-4, which may facilitate more refined functions of CTLA-4 within the mammalian immune system.

## Introduction

Within the immune system, “co-stimulation” via the CD28 receptor permits robust and effective CD4^+^ T cell responses important for effective immunity. This is mediated by binding to two ligands CD80 and CD86. Critically, a second receptor, CTLA-4, also binds these ligands but acts as a negative regulator of T cell responses, effectively preventing CD28 co-stimulation. Mice deficient in CTLA-4 die of autoimmune organ destruction mediated by CD4^+^ T cells highlighting the essential role of this pathway in immune regulation [1], [2]. Thus the interactions between CD28, CTLA-4 and their ligands dictate essential functions during activation of the T cell response. Whilst CD28 is robustly expressed on the T cell surface, CTLA-4 is constitutively internalised from the plasma membrane and at steady state, is predominantly located in intracellular compartments raising the question of how intracellular trafficking might affect the function of CTLA-4.

It is known that CTLA-4 internalisation is mediated by the interaction of a tyrosine-based endocytic motif located within the cytoplasmic C-terminus of CTLA-4 [3]–[6]. This interacts with the clathrin adaptor AP-2 to mediate clathrin-dependent endocytosis [3]–[6]. Following internalisation, CTLA-4 is then either degraded in lysosomal compartments or recycled back to the plasma membrane [4], [7], [8]. We recently proposed that this endocytic ability may play an important role in CTLA-4 function by facilitating the capture of its transmembrane co-stimulatory ligands from opposing cells by a process of transendocytosis [9]. According to this model CTLA-4 is able to regulate the function of CD28 by depleting antigen presenting cells of ligands thereby preventing CD28 engagement and signaling. Moreover, this transendocytosis function of CTLA-4 is seen on both regulatory T cells and non-specialized T cells that express CTLA-4.

We were therefore interested in the extent to which CTLA-4 intracellular trafficking is conserved during evolution. The CD28/CTLA-4 co-stimulatory system appears to be present in jawed vertebrates and CTLA-4 has been identified in teleost fish, amphibians, birds and mammals [10]–[12]. Features of the extracellular ligand-binding site such as M(/L)YPPPY motif appear to be well conserved across species. In contrast, whilst the cytoplasmic domain shows a striking degree of conservation in mammals it is much less well conserved in non-mammals. In this study we have investigated the ability of different CTLA-4 C-termini from non-mammals to direct intracellular trafficking using chimeric proteins composed of the human CTLA-4 ectodomain fused with the cytoplasmic tail of chicken, *xenopus* or trout. Our results reveal considerable variation in CTLA-4 internalisation kinetics and recycling between species, which support the concept that intracellular trafficking has evolved as part of the refinement of CTLA-4 function.

## Results

### Comparison of the intracellular distribution of CTLA-4 orthologues

**Figure 1:**
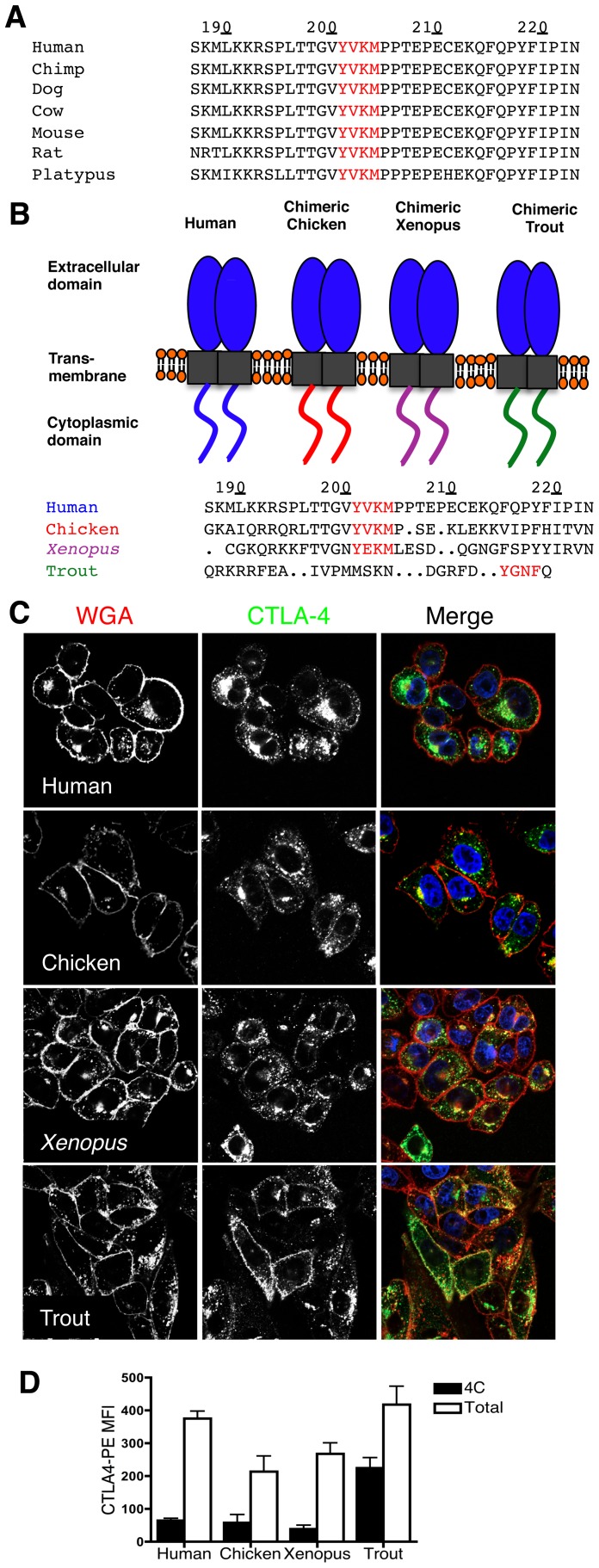
Generation and localisation of CTLA-4 chimeras. **A**. C-terminal sequence alignments of selected mammalian CTLA-4 based on sequence data from Ensembl and in ref 12. **B.** Diagram of human CTLA-4 chimeras containing the extracellular and transmembrane domain of human CTLA-4 and the C-terminus of species shown. C-terminal amino acid sequence alignments of human, chicken, *xenopus* and trout CTLA-4 are shown below, based on alignments using Clustal W. **C**. CHO cells expressing CTLA-4 chimeras were incubated with WGA-tetramethylrhodamine at 4°C for 45minutes. Cells were subsequently fixed, permeabilised, and stained with an unlabeled anti-CTLA-4 Ab followed by Alexa488 anti-human IgG (green) to stain total CTLA-4 protein. Cells were analysed by confocal microscopy. **D** Relative expression of surface (4°C) and total CTLA-4 for each chimera as determined by flow cytometry.

In mammals, there is essentially 100% amino acid sequence conservation of the CTLA-4 cytoplasmic tail **(** **Figure 1A** **)** [10]–[12]. This supports the view that any protein sorting signals encoded within this region are likely to be of functional importance. In contrast, in species such as chicken, *xenopus* and trout, there is considerable variation in this region **(** **Figure 1B** **)**, which may provide insights into CTLA-4 trafficking and the regulation of co-stimulation. We therefore generated chimeric versions of human CTLA-4 where the C-terminus was replaced with that of chicken, *xenopus*, or trout CTLA-4 **(** **Figure 1B** **)**. We transfected Chinese hamster ovary (CHO) cell lines with the CTLA-4 chimeras, labeled the cell surface with wheat germ agglutinin (WGA) at 4°C and then stained cells for total CTLA-4 expression using an antibody to the human CTLA-4 ectodomain to assess localisation **(** **Figure 1C** **)**. *Xenopus* and chicken chimeras revealed a pattern similar to human CTLA-4 with a punctate intracellular distribution. In contrast, the chimera with the trout C-terminus showed robust surface expression with far more limited intracellular vesicles. This difference in the amount of surface CTLA-4 relative to the total was quantified by flow cytometry and is shown in **figure 1D**.

### Comparison of the endocytic ability of CTLA-4 orthologues

**Figure 2:**
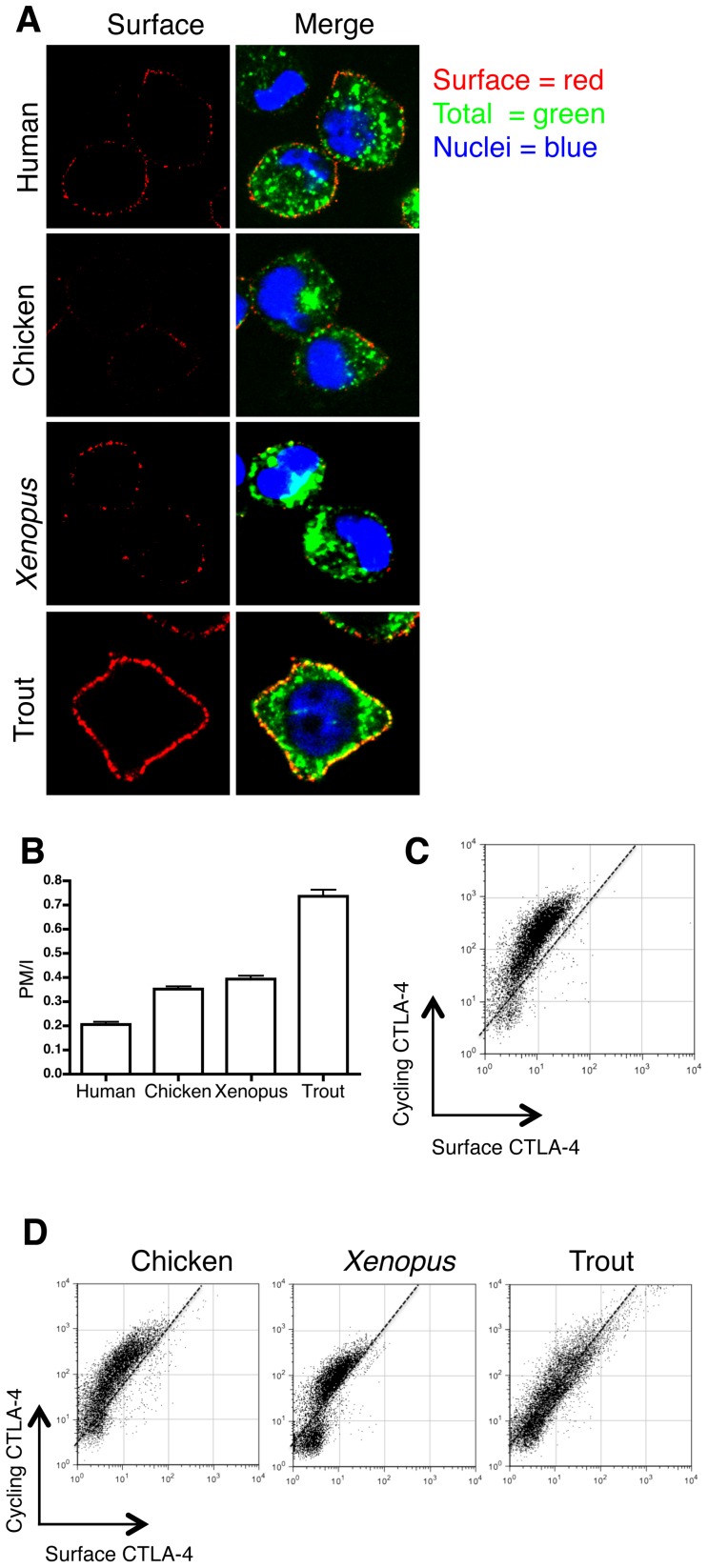
Cellular localisation of CTLA-4 chimeras. **A.** CHO cells expressing CTLA-4 chimeras were incubated with unlabeled anti-CTLA-4 Ab at 37°C for 1 hour, cooled to 4°C and surface CTLA-4 stained red with anti-mouse Alexa 555. Cells were subsequently fixed, permeabilised and stained with Alexa488 anti-mouse IgG (green) and imaged by confocal microscopy. **B.** The ratio of plasma membrane to internalised CTLA-4 fluorescence (PM/I) was calculated by outlining cells in ImageJ. **C.** CHO cells expressing human CTLA-4 were labeled with anti-CTLA-4 PE at 37°C for 30 minutes followed by labeling surface CTLA-4 on ice (4°C) with Alexa647 anti-mouse IgG. Cells were analysed by flow cytometry and data are plotted as cycling CTLA-4 (37°C label) vs surface CTLA-4 (4°C label). **D.** CHO cells expressing the CTLA-4 chimeras were labeled as described in **C** and analysed by flow cytometry. Dotted line provides a standard gradient for reference purposes.

The increased surface expression observed with chimeric trout CTLA-4 suggested that the C-terminus of trout CTLA-4 might confer less efficient internalisation consistent with its lack of a YXKM motif. To assay internalisation directly, we labeled cells at 37°C with an unconjugated anti-CTLA-4 Ab so as to label CTLA-4 protein cycling from the plasma membrane. Cells were subsequently placed on ice to prevent further trafficking and receptors remaining at the cell surface labeled with a fluorescently conjugated secondary antibody (red). Cells were then fixed and permeabilised and internalised CTLA-4 protein detected with a different fluorescently conjugated secondary antibody (green) before analysing cells by confocal microscopy **(** **Figure 2A** **)**. To quantify these differences, internalisation was also measured as a ratio of plasma membrane (red) to internalised (green) CTLA-4 **(** **Figure 2B** **)**. Human CTLA-4 possessed the lowest surface to internalised ratio reflecting that CTLA-4 is predominantly localised in intracellular vesicles, which was similar to chimeric constructs from *xenopus* and chicken CTLA-4. In contrast, the C-terminus of trout CTLA-4 showed a greater surface to internalised ratio suggesting relatively poor endocytosis consistent with its more obvious surface phenotype.

To assay the efficiency of CTLA-4 internalisation more quantitatively in multiple cells, we used a flow cytometric approach. Cycling CTLA-4 was labeled with a PE-conjugated anti-CTLA-4 Ab at 37°C. Cells were subsequently washed and placed on ice and any residual surface primary antibody was detected using a secondary antibody **(** **Figure 2C** **)**. For WT CTLA-4 this generates a curved plot where the extensive cycling label at 37°C (Y-axis) is greater than the minimal surface label (x-axis), typical of an endocytic protein. Whilst both chicken and *xenopus* CTLA-4 chimeras showed a similar pattern to human the chimeric trout CTLA-4 displayed an almost linear relationship between cycling and surface CTLA-4 **(** **Figure 2D** **)** typical of a cell surface protein, again suggesting impaired CTLA-4 endocytosis in trout.

**Figure 3:**
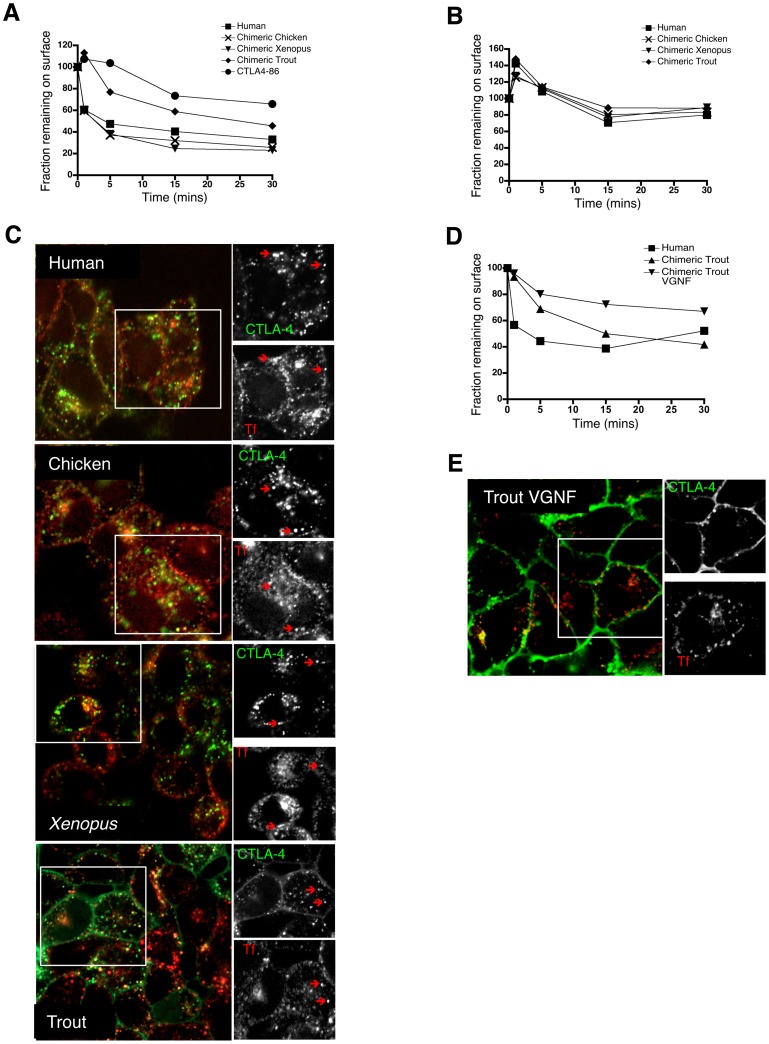
Endocytosis rates of CTLA-4 chimeras. **A.** CHO cells expressing CTLA-4 chimeras were labeled at 4°C with anti-CTLA-4 to label surface CTLA-4. Cells were then warmed to 37°C to allow endocytosis for the times indicated. Cells were then placed on ice and any remaining surface CTLA-4 detected with Alexa647 anti-mouse IgG. The 647 signal was plotted against time as the fraction remaining compared to 4°C. **B.** CHO cells expressing CTLA-4 chimeras were labeled as in A but in medium supplemented with sucrose (0.45 M) to prevent endocytosis. **C.** CHO cells expressing the chimeric CTLA-4 constructs were incubated with a transferrin (Tf) Alexa633 conjugate (Invitrogen) and anti-CTLA-4 PE at 37°C for 45 minutes. Cells were subsequently fixed and analysed by confocal microscopy. The red arrows indicate co-localisation. **D.** Rate of endocytosis of VGNF mutant was performed as described in A. **E.** Transferrin uptake of VGNF mutant was performed as described in C and analysed by confocal microscopy.

To directly measure the rates of endocytosis we stained the cell surface pool of CTLA-4 on ice with an unconjugated antibody. Cells were then warmed to 37°C for the indicated time-points to allow any internalisation of surface CTLA-4 to take place. Cells were then placed on ice, and any CTLA-4 remaining at the cell surface detected with an Alexa647 conjugated secondary antibody. Accordingly, in this assay the loss of Alexa647 staining over time reflects endocytosis of CTLA-4. Using this assay, human CTLA-4 showed a comparable rate of endocytosis to the chimeric *xenopus* and chicken constructs **(** **Figure 3A** **)**, internalising 50% or more within 5 minutes. In contrast, chimeric trout CTLA-4 showed a much slower rate of endocytosis taking at least 30 minutes to achieve 50% internalisation. Nevertheless, chimeric trout CTLA-4 did internalise compared to a control CTLA-4 chimera with a cytoplasmic domain from CD86, which is a plasma membrane resident in CHO cells **(** **Figure 3A** **)**. However, it was clear that even for surface proteins (CTLA-4-CD86) there was a small decrease in signal over time in this assay, which was not due to endocytosis, further emphasising the much reduced endocytic nature of the trout chimera.

Since internalisation of CTLA-4 occurs via an AP-2 mediated, clathrin-dependent pathway [3], [5], [6], treatment with hypertonic sucrose can be used to inhibit the formation of clathrin-coated vesicles [13]. We therefore tested the effect of sucrose treatment on human CTLA-4 and the chimeric constructs. As shown in **figure 3B** sucrose treatment inhibited the endocytosis of CTLA-4 molecules. In particular the more rapid endocytosis seen in chicken, *xenopus* and human CTLA-4 chimeras (relative to trout) was prevented by sucrose treatment. Overall, this data suggests that the chimeras internalise via a clathrin dependent pathway.

The transferrin receptor is well characterized and internalizes by clathrin-dependent endocytosis using signals encoded in the cytoplasmic tail [14]. Using transferrin as a marker for the clathrin pathway, we compared the co-localisation of the CTLA-4 chimeras with transferrin. Cells were incubated with transferrin AlexaFluor633 and anti-CTLA-4 PE at 37°C for 45 minutes and subsequently fixed and analysed by confocal microscopy. This revealed that human, chicken and *xenopus* CTLA-4 co-localised with transferrin in intracellular vesicles, suggesting CTLA-4 internalisation overlaps with the transferrin receptor consistent with both proteins utilizing the clathrin-dependent pathway **(** **Figure 3C** **)**. Additionally, this assay revealed limited but detectable co-localisation between trout CTLA-4 and transferrin, further suggesting that trout CTLA-4 does internalise via clathrin albeit at a reduced rate.

Whilst trout CTLA-4 lacked the conserved YVKM internalisation motif found in mammals, it did appear to have a putative YxxF motif [12], which could possibly mediate endocytosis. We therefore tested whether this motif contributed to the internalisation observed with the chimeric trout CTLA-4. We mutated this tyrosine residue to a valine and examined the behaviour of this mutant in CHO cells. A comparison of internalisation rates indicated the VGNF mutant had further impaired endocytosis compared to the YGNF control and now resembled the behavior of non-endocytic proteins seen above using sucrose or with the surface CD86 chimera **(** **Figure 3D** **)**. In contrast to chimeric trout CTLA-4 containing YGNF, the VGNF mutant demonstrated no co-localisation with transferrin **(** **Figure 3E** **)**. Together, these results confirmed the presence of a functional tyrosine-based endocytic motif in trout CTLA-4 albeit one that functions with reduced efficiency.

### Degradation of CTLA-4 orthologues correlates with endocytic ability

**Figure 4:**
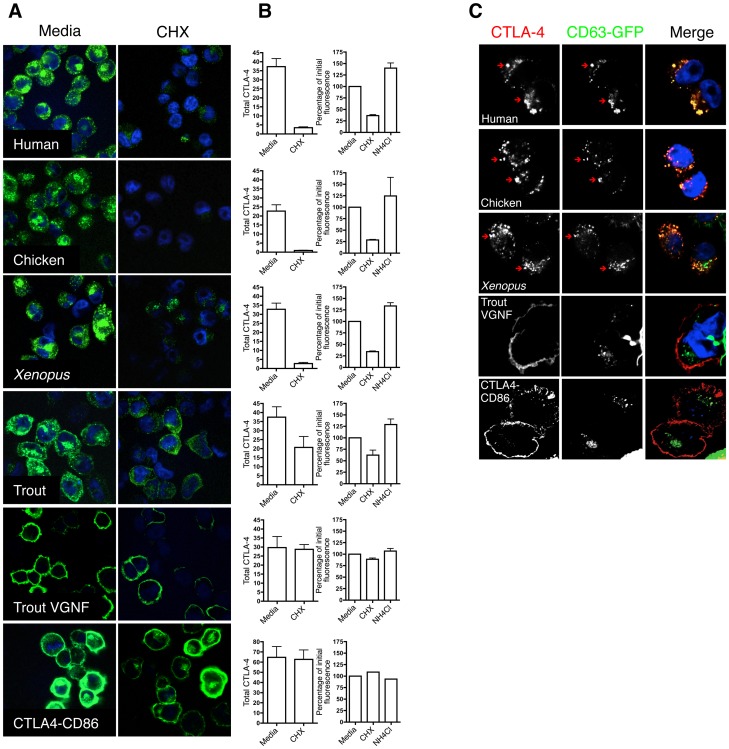
Degradation of CTLA-4 chimeras. **A**. CHO cells expressing CTLA-4 chimeras were incubated in medium or medium supplemented with cycloheximide (CHX) at 37°C for 3 hours. Cells were fixed, permeabilised and stained for total CTLA-4 with an unlabeled anti-CTLA-4 Ab followed by Alexa488 anti-mouse IgG (green) and nuclei counterstained using DAPI (blue). Cells were analysed by confocal microscopy. **B** Total CTLA-4 was quantified by outlining cells in ImageJ and MFI plotted (left column). For flow cytometric quantitation (right hand column) cells were stained for total CTLA-4 using anti-CTLA-4 PE after 3 hours of CHX or NH_4_Cl treatment at 37°C and MFI plotted as a percentage of initial fluorescence. **C**. CHO cells expressing CTLA-4 chimeras were transfected with CD63-GFP. Cells were incubated in medium supplemented with NH_4_Cl at 37°C for 3 hours. Cells were fixed, permeabilised, and stained with an unlabeled anti-CTLA-4 Ab and Alexa565 anti-human IgG (red) to stain total CTLA-4 protein and analysed by confocal microscopy. The red arrows indicate co-localisation.

CTLA-4 has previously been reported to interact with the lysosomal sorting adaptor AP-1 and to undergo degradation in lysosomal compartments [8], [15]. We therefore compared the stability of our CTLA-4 chimeras by blocking new protein synthesis using cycloheximide (CHX) and monitoring the decay of existing CTLA-4. In addition, if CTLA-4 was being degraded via a lysosomal pathway then ammonium chloride (NH_4_Cl) should prevent degradation. We therefore monitored CTLA-4 protein stability in the presence of CHX or NH_4_Cl for 3 hours at 37°C. After treatment, cells were fixed and permeabilised prior to staining to reveal total CTLA-4 expression and analysed by confocal microscopy or flow cytometry. In the absence of new protein synthesis, rapid loss of human, chicken and *xenopus* CTLA-4 was observed **(** **Figure 4A** **)** indicating that CTLA-4 was degraded rapidly. Degradation was quantified by both confocal analysis **(** **Fig. 4B** **–left column)** and by flow cytometry **(** **Fig. 4B** **–right column)**. Moreover, NH_4_Cl resulted in an accumulation of CTLA-4 (predominantly in human, xenopus and chicken chimeras) suggesting that blocking lysosomal function prevents CTLA-4 degradation **(** **Figure 4B** **)**. Whilst human CTLA-4, chimeric *xenopus* and chicken showed comparable degradation, the trout CTLA-4 chimera was much less affected by CHX although compared to the non-endocytic variants (trout VGNF and CTLA-4-CD86 chimera) some degradation was still evident **(** **Figure 4B** **)**.

To determine if CTLA-4 co-localised with markers of lysosomes, the CTLA-4 chimeras were also transfected with the lysosomal membrane protein CD63, fused to GFP, in the presence of NH_4_Cl. Notably, human, chicken and *xenopus* CTLA-4 all demonstrated substantial co-localisation with CD63-GFP, suggesting traffic to lysosomal compartments **(** **Figure 4C** **)**. In contrast, the non endocytic trout VGNF mutant and the cell surface CTLA-4-CD86 chimera demonstrated no obvious co-localisation with CD63. Collectively these data suggest that degradation of CTLA-4 is related to its endocytic capacity and that the presence of YV/EKM motif promotes both internalisation and sensitivity to lysosomal degradation. Moreover, trout CTLA-4 contains an alternate tyrosine-based motif that has a reduced efficiency of endocytosis and has a more stable cell surface phenotype when compared to CTLA-4 from human, chicken and *xenopus*.

### Differing ability to recycle amongst CTLA-4 orthologues

**Figure 5:**
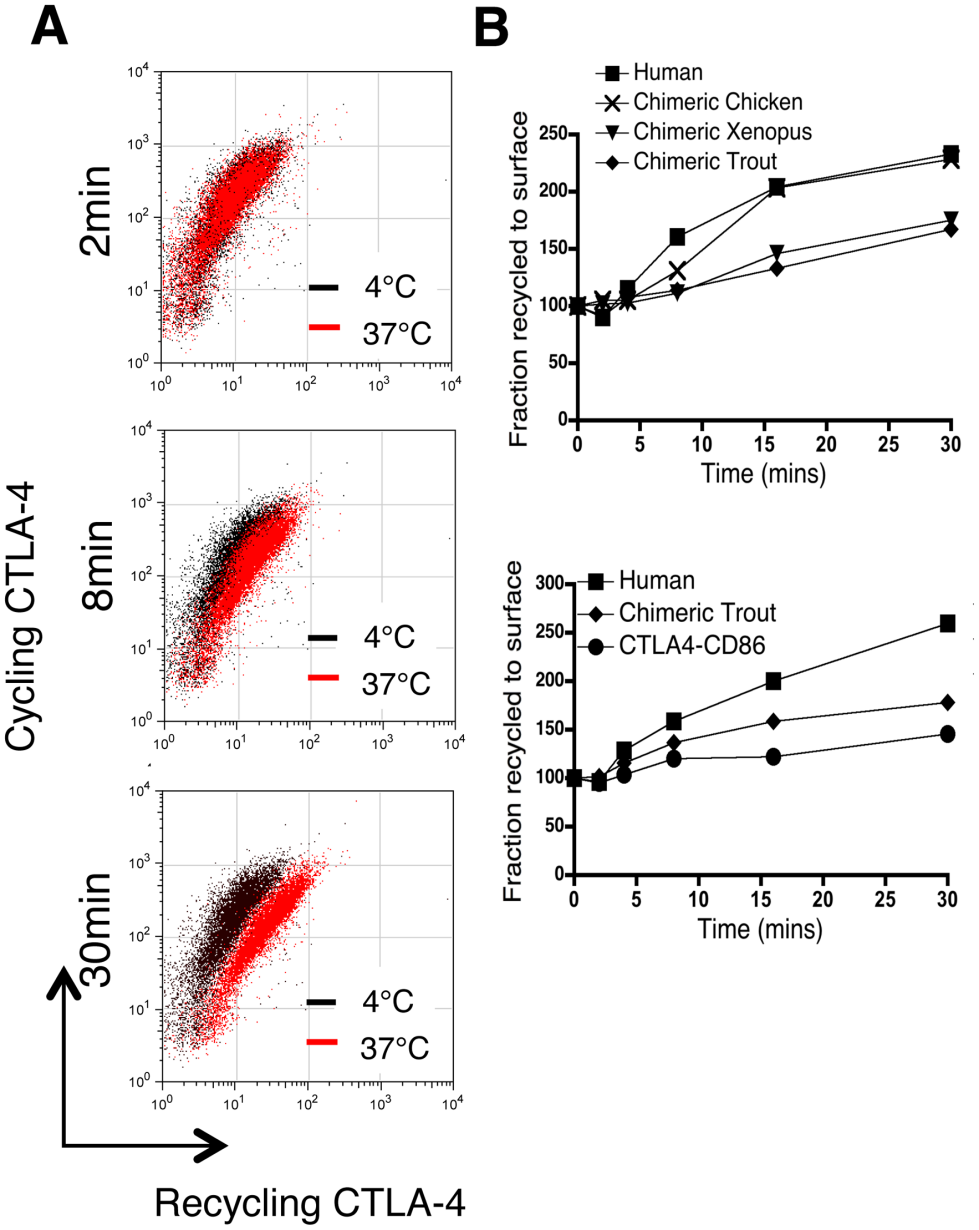
Recycling rates of CTLA-4 chimeras. **A**. CHO cells expressing WT human CTLA-4 were labeled with mouse anti-CTLA-4 PE at 37°C to detect cycling CTLA-4, washed and any recycling CTLA-4-PE antibody detected by addition of Alexa647 anti-mouse IgG at either 4°C or 37°C. Representative FACS plots are shown for PE-label (cycling CTLA-4) vs Alexa647 label (re-cycling CTLA-4) at the indicated time points. **B**. Recycling rates are plotted for the chimeras as normalised to the 4°C control.

We have recently shown that once internalised a proportion of CTLA-4 molecules can recycle back to the plasma membrane [4]. Since variation in endocytic motifs may also affect recycling [16], we assessed this property in our chimeras. To assay CTLA-4 recycling, cells were incubated at 37°C with a PE-conjugated anti-CTLA-4 Ab for 30 minutes to label the cycling pool of CTLA-4. Cells were then incubated further at 37°C for various times with an Alexa647 antibody to label any of the primary antibody recycling back to the cell surface. Accordingly, recycling CTLA-4 is seen as an increase in the Alexa647 signal over time. Notably, human CTLA-4 and chimeric chicken CTLA-4 (YVKM containing proteins) showed a comparable rate of recycling **(** **Figure 5A and B** **)** whereas chimeric *xenopus* and trout CTLA-4, which lacked YVKM motif were notably less efficient.

**Figure 6:**
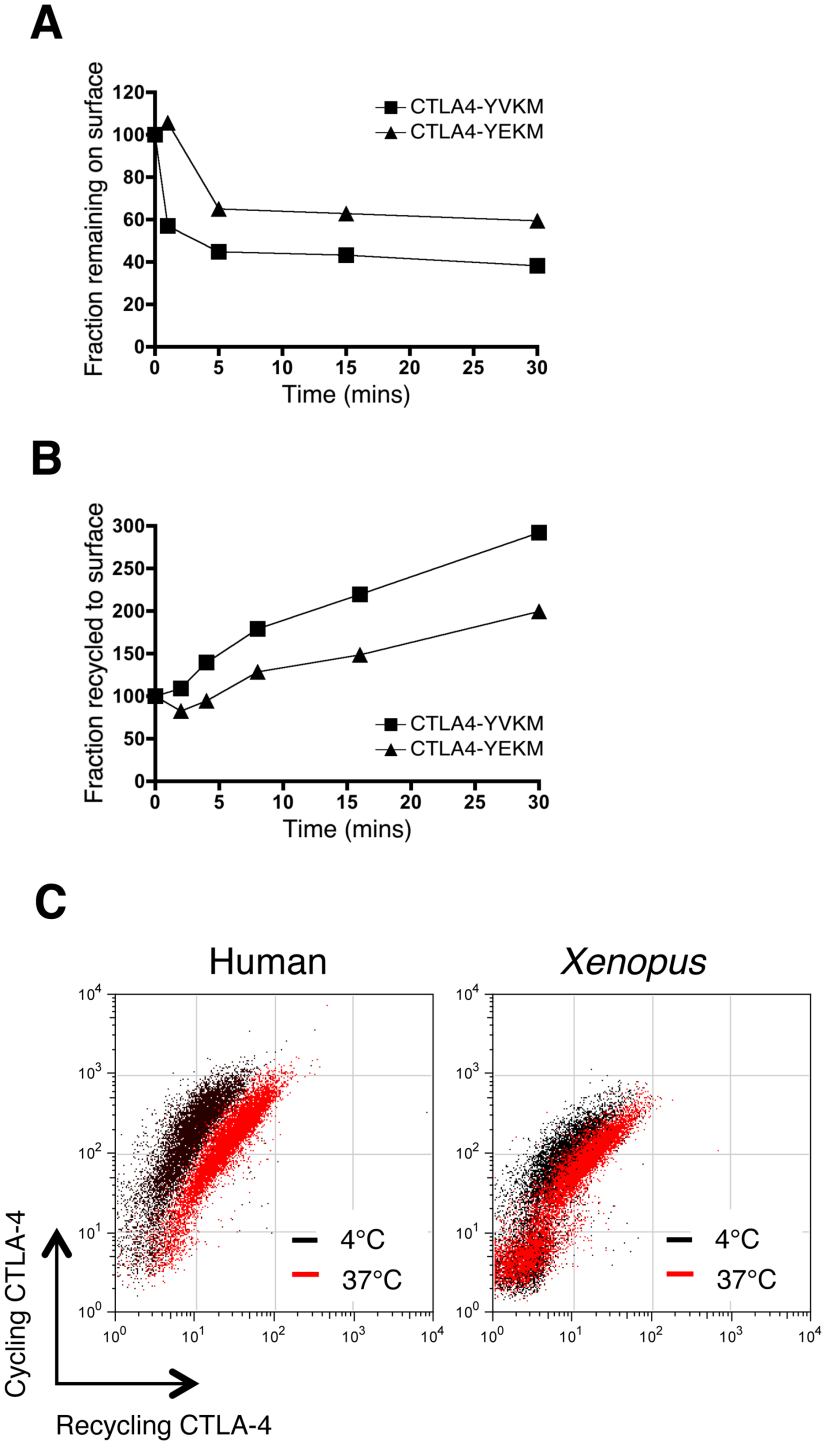
Recycling efficiency is regulated by the YVKM motif. **A**. CHO cells expressing WT human CTLA-4 YVKM or YEKM motif were labeled at 4°C with anti-CTLA-4 to label surface CTLA-4. Cells were warmed to 37°C for the time indicated. Cells were then placed on ice and the remaining surface CTLA-4 detected with Alexa647 anti-mouse IgG and plotted over time. **B**. CHO cells expressing WT human CTLA-4 YVKM or a point mutant YEKM motif were labeled with mouse anti-CTLA-4 PE at 37°C to detect cycling CTLA-4. Recycling protein was detected with Alexa647 anti-mouse IgG at 37°C for the indicated time points. Recycling rates are plotted for the CTLA-4 variants normalised to the 4°C control. **C**. Representative data comparing human and *xenopus* CTLA-4 recycling after 30 minutes is shown.

Since an acidic residue (E) at the +1 position of YxxM has been suggested to favour an interaction with the lysosomal sorting adaptor AP-3 [17], we mutated the YVKM motif in human CTLA-4 to YEKM and assayed the efficiency of internalisation and recycling. The CTLA-4 YEKM construct remained endocytic albeit with slightly reduced efficiency compared to CTLA-4 YVKM **(** **Figure 6A** **)**. However, we also observed impaired recycling by CTLA-4 YEKM **(** **Figure 6B** **)**, which was reminiscent of the lower recycling observed for the *xenopus* CTLA-4 chimera **(** **Figure 6C** **)**. Taken together these data suggest that the YVKM motif found in mammalian CTLA-4 is optimised for both endocytosis and recycling of CTLA-4 and that even relatively subtle variations as found in *xenopus* CTLA-4 can compromise these functions.

## Discussion

The role of intracellular trafficking in CTLA-4 function is not well understood, however the high degree of cytoplasmic domain sequence conservation in mammals is intriguing. CTLA-4-expressing T cells play an essential role in immune regulation where CTLA-4 is required in order to prevent T cell responses to host tissues. This is achieved by expression of CTLA-4 on specialised regulatory T cells [18]. We have recently shown that a core feature of CTLA-4 biology is its ability to capture ligands from opposing cells, internalise them in a process termed transendocytosis [9]. Such a function presumably involves the specialised intracellular trafficking itinerary. Indeed it is clear that ligands captured by transendocytosis are degraded in the lysosomes in the CTLA-4 expressing cells [9]. Here we have analysed the impact of naturally occurring C-terminal sequence variations of CTLA-4 (found in non-mammals) for their impact on trafficking.

We show that C-terminal amino acid sequence variation has an impact on CTLA-4 endocytosis, recycling and degradation. Whilst in general CTLA-4 shows the ability to internalise in all the chimeras tested, we noted that the C-terminus of trout CTLA-4 conferred a substantially reduced efficiency and consequently a much higher plasma membrane expression at steady state. Nonetheless endocytosis could occur through a newly identified YGNF motif. Interestingly this YxxF motif is found in several fish species including trout and salmon. Moreover, in zebrafish a YVKF motif is observed which is remarkably similar to the YVKM found in mammalian CTLA-4 [12]. Whilst there are a number of fish genomes undergoing analysis, at present the unequivocal identification of CD28 and CTLA-4 genes is still limited and therefore awaits further characterization. Thus it is plausible that these motifs represent the initial, albeit rather ineffective, emergence of endocytosis in CTLA-4. In contrast to fish, the C-terminus of amphibian (*Xenopus*) and chicken (*Gallus gallus*) CTLA-4 both conferred strong endocytic ability that was more similar to that of human CTLA-4. This endocytic ability was also well correlated with the susceptibility of CTLA-4 to degradation suggesting endocytosis exposes CTLA-4 to degradation in lysosomes. We also observed some differences in recycling ability of CTLA-4 between various species. In particular, differences between human and *xenopus* CTLA-4 appeared to relate to the use of the less efficient YEKM by *xenopus*. Taken together our data show that CTLA-4 C-terminal domain directs a relatively complex intracellular trafficking itinerary, which is to a large extent seen in amphibians and birds, albeit with some variation. In contrast, these features are much less well developed in fish suggesting that CTLA-4 function may have evolved via mutation and selection of cytoplasmic domain variants.

It is interesting to speculate on the impact of these changes in trafficking on CTLA-4 function. It is known that the major innovations that have taken place in vertebrate immune systems relate to the development of lymph nodes [19] and more specialised immune features such as T cell memory enhanced effector function. These features are found in mammals and birds but not in fish. Interestingly, such advanced immune features have also given rise to the requirement for a subset of specialised regulatory T cells which prevent immune damage to self tissues possibly required to regulate these enhanced effector functions [20]. It is interesting to note that CTLA-4 is utilized as a major effector molecule expressed by regulatory T cells [2]. Thus one might tentatively suggest that in order to operate in a cell-extrinsic manner (required by regulatory T cells) CTLA-4 [18]internalization and intracellular trafficking may have been adapted in order to facilitate efficient ligand removal and disposal from antigen presenting cells. In contrast, in species without the need for such specialised regulation, CTLA-4 may have been able to perform useful functions by competing for ligand binding whilst remaining predominantly at the cell surface as seen in fish CTLA-4.

## Materials and Methods

### DNA constructs and transfectants

Full-length CTLA-4 cDNA was cloned into a CMV expression vector pcDNA3.1 as previously described [21]. Chimeric proteins of human CTLA-4 with the cytoplasmic tail of chicken, xenopus or trout were synthesised by Genscript and cloned into the same vector. Point mutation of CTLA-4 YEKM and Human Trout VGNF CTLA-4 chimera were generated using the QuikChange Lightning Site-Directed Mutagenesis Kit (Agilent Technologies).

### Cell culture and tissue culture

Chinese hamster ovary (CHO) cells were cultured in DMEM medium supplemented with 2 mM L-glutamine, 10% FBS, 1% penicillin and streptomycin in a humidified 37°C/5% CO_2_ incubator and passaged by trypsinisation. CHO cell lines expressing different cDNA constructs were generated by electroporation (AMAXA). Cells expressing the CTLA-4 chimeras were selected using G418 (500 µg/ml) treatment and by cell sorting.

### Confocal microscopy

Imaging was carried out using a Zeiss Axiovert LSM510 confocal microscope and Zeiss LSM 780 inverted laser scanning confocal microscope using a 100X oil-immersion objective with excitation at 488 nm, 543 nm and 633 nm. Constant laser powers and acquisition parameters were maintained throughout individual experiments for analysis. For quantitation, cells were outlined and mean fluorescence intensity (MFI) measured using ImageJ (Wayne Rasband, NIH). All confocal images shown are representative of at least 40 cells taken from at least three independent experiments.

For analysis of localisation, CHO cells expressing CTLA-4 chimeras were plated overnight on a poly-L-lysine coated coverslip in a 24 well plate. For surface expression, cells were incubated with Wheat Germ Agglutinin (WGA) - tetramethylrhodamine conjugate (Molecular Probes, Invitrogen) at 4°C for 45 minutes. Cells were then washed with 4°C medium and fixed in 3% PFA in PBS for 15 minutes. Cells were permeabilised with PBS containing 0.1% saponin (PBS/sap) and receptors detected by incubation with PBS/sap containing unconjugated anti-CTLA-4 Ab and Alexa488 conjugated anti-human IgG. Following staining, coverslips were dried, inverted and mounted with Vectorshield (Vector Laboratories, UK) prior to visualisation by confocal microscopy.

For analysis of transferrin uptake, CHO cells expressing CTLA-4 chimeras were incubated at 37°C with Transferrin AlexaFluor633 conjugate (Molecular Probes, Invitrogen) and anti-CTLA-4 PE at 37°C for 45 minutes. Cells were then washed with 4°C medium and fixed in 3% PFA in PBS.

For analysis of surface vs internalised CTLA-4, CHO cells expressing CTLA-4 chimeras were incubated at 37°C with unconjugated anti-CTLA-4 Ab (clone 11G1) for 1 hour. Cells were then washed 3 times in medium (4°C) and placed on ice. Surface receptors were labeled on ice by addition of Alexa555 conjugated anti-mouse IgG. Cells were then washed with 4°C medium and fixed in 100% Methanol at −20°C for 30 minutes. Internalised receptors were then detected by incubation with PBS containing Alexa488 conjugated anti-mouse IgG.

For analysis of degradation, cells were incubated in medium alone or medium supplemented with CHX at 37°C for 3 hours. Cells were fixed with 3% PFA in PBS and permeabilised with PBS/sap. Total CTLA-4 was then detected by incubation with unconjugated anti-CTLA-4 Ab (clone 11G1) and Alexa488 conjugated anti-mouse IgG in PBS/sap.

For analysis of CTLA-4 co-localisation with the lysosomal pathway, CHO cells expressing CTLA-4 chimeras were transfected with CD63-GFP and incubated in medium supplemented with NH_4_Cl to prevent CTLA-4 degradation. Total CTLA-4 was then detected by incubation with unconjugated anti-CTLA-4 Ab followed by Alexa565 conjugated anti-human IgG in PBS/sap.

### Flow Cytometric Analysis

Flow cytometry was carried out using a FACScan flow cytometry (BD Biosciences – Oxford, UK) and acquired using CellQuest software. Analysis was performed using FlowJo (TreeStar). Flow cytometric plots are representative of at least three independent experiments.

For analysis of localisation, cells were incubated with anti-CTLA-4 PE (BD Biosciences) for 30minutes at 4°C (surface) or were fixed and permeabilised and stained for total CTLA-4. Cells were analysed by flow cytometry and MFI plotted.

For analysis of internalisation, cells were incubated in medium or medium supplemented with 0.45 M Sucrose and labeled with anti-CTLA-4 PE (BD Biosciences) for 30 minutes at 4°C. Cells were then placed on ice and washed in 4°C medium or sucrose. Cells were raised to 37°C for various time points, placed on ice and were stained for surface CTLA-4 with Alexa647 conjugated anti-mouse IgG. Cells were then washed in 4°C medium and analysed by flow cytometry.

For analysis of recycling, cells were incubated with anti-CTLA-4 PE for 30 minutes at 37°C. Cells were then placed on ice and washed in 4°C medium. Surface CTLA-4 was then labeled by incubation on ice with Alexa647 conjugated anti-mouse IgG. Recycling CTLA-4 was labeled by incubation at 37°C with Alexa647 conjugated anti-mouse IgG for various time points. Cells were then placed on ice and washed in 4°C medium.

For analysis of degradation, cells were incubated in medium or medium supplemented with CHX or NH_4_Cl at 37°C for 3 hours. Cells were fixed with 3% PFA in PBS and permeabilised with PBS/sap. Total CTLA-4 was then detected by incubation with anti-CTLA-4 PE.
